# American Crows as Carriers of Extra Intestinal Pathogenic *E. coli* and Avian Pathogenic-Like *E. coli* and Their Potential Impact on a Constructed Wetland

**DOI:** 10.3390/microorganisms8101595

**Published:** 2020-10-16

**Authors:** Keya Sen, Vaughn Shepherd, Tanner Berglund, Alexa Quintana, Shnia Puim, Rama Tadmori, Robert J. Turner, Laura Khalil, Marilia A. Soares

**Affiliations:** 1 Division of Biological Sciences, STEM, University of Washington, Bothell, WA 98011, USA; vaughn94@uw.edu (V.S.); tanner.berglund@gmail.com (T.B.); alexaquintana@gmail.com (A.Q.); puimshnia@gmail.com (S.P.); tadmor@uw.edu (R.T.); kLaura24@hotmail.com (L.K.); marilia.89@hotmail.com (M.A.S.); 2 School of Interdisciplinary Arts & Sciences, University of Washington, Bothell, WA 98011, USA; rturner1@uw.edu

**Keywords:** environmental water, virulence, antibiotic, *E. coli*, wetland, corvids, ExPEC, APEC, zoonosis

## Abstract

The study examines whether crows are carriers of extraintestinal pathogenic *E. coli* (ExPEC) and avian pathogenic *E. coli* (APEC)-like strains, and if wetland roost areas contribute to their spread. A total of 10 crow feces (*n* = 71) and 15 water *E. coli* isolates (*n* = 134) from a wetland area could be characterized as potentially ExPEC based on the presence of ≥2 of the five cardinal genes *iutA, kpsMT2, papEF, pap A/C, papG, sfa/foc,* and *afa/dra,* while six fecal and 14 water isolates could be characterized as potentially APEC-like based on the presence of plasmid associated genes: *iutA*, episomal *iss, ompT, hlyF* and *iroN*. A total of 32 fecal and 27 water isolates tested carried plasmids based on incompatibility typing. Plasmids from 34 of 38 isolates tested could be transferred to another *E. coli* strain by conjugation with the antibiotic resistance (AR) profile being transferred, indicating their potential to be transferred to indigenous and non-pathogenic strains in the wetland. APEC-like plasmids could be transferred in six of eight isolates tested. Pathogenic *E. coli* of importance to the medical community and poultry industry may be detected in high levels in surface water due to corvid activity. Regardless of their role in health or disease, water in wetlands and streams can serve as a media for the dissemination of AR and virulence traits of bacteria, with corvids acting as potential vectors for farther dissemination.

## 1. Introduction

Wild birds, including crows, can act as reservoirs and also be vectors in the spread of pathogens that are capable of causing diseases in both humans and animals [1,2]. Several bacterial and viral infections have been linked to crows, including infection by West Nile Virus in both humans and avian species, human diarrhea due to *Salmonella* spp., *Shigella* spp., and *E. coli,* and sporadic cases of campylobacteriosis [2,3,4,5,6]. Their feeding, foraging habits, and migratory behavior, can contribute to their ability to spread pathogens [2]. 

The gram-negative bacteria *E. coli* present in the gut of most mammals and birds are primarily harmless commensals, though certain strains can be capable of causing severe infections [7]. Since *E. coli* can also survive a wide range of environmental conditions outside of the gut, with some strains being more adaptable than others to a given environment, it gives them an opportunity to be spread even farther than their original habitat [8]. The pathogenic strains have a set of virulence factors, and depending on the type and combination of virulence factors present, can cause a variety of infections. Broadly, those strains causing gastrointestinal diseases belong to the intestinal pathogenic *E. coli* (IPEC) pathotype, while those causing extraintestinal diseases are of the extraintestinal pathogenic *E. coli* (ExPEC) pathotype. ExPEC pathotypes include uropathogenic *E. coli* (UPEC), neonatal meningitis *E. coli* (NMEC), and avian pathogenic *E. coli* (APEC) that cause avian colibacillosis [9,10]. 

The different subtypes of ExPEC have been shown to carry virulence factor genes that are very similar, although they may come from different sources, hosts, and tissues [9,11,12]. Whole genome sequencing of one APEC O1 strain showed 95.5% of the genome was present in other sequenced human ExPEC genomes [13], and it was suggested that APEC strains from poultry and other meat products could serve as a foodborne source for ExPEC disease in humans either directly by contamination or as a reservoir of virulence genes that get transferred to human commensals [14]. Other studies that performed comparative genomic analysis showed that an APEC O2:K1 strain (IMT5155) shared significant overlap/similarities with APEC O1 as well as to two human ExPEC O18:K1 strains [12,15]. The regions of homology included various virulence determinants that are needed for establishing infection in a host [15]. Pathogenicity tests of these APEC O1:K1 and O2:K1 serotypes, as well as several other APEC and NMEC O18 strains in animal models, showed them to be highly virulent for avian colisepticemia, and also be able to cause septicemia and meningitis in neonatal rats [12,15]. These findings indicate that the distribution of APEC O1:K1 and O2:K1 serotypes in the environment might pose a significant zoonotic threat. 

Extensive statistical analysis of virulence factor (VF) genes from three strain collections, which had earlier been shown to be causative for ExPEC related diseases or infections in animal models, has established that a strain must possess ≥2 of the following five virulence marker genes to be classified as ExPEC: *pap* (P fimbriae), *sfa/foc* (S/F1C fimbriae), *afa/dra* (Dr binding adhesins), *iutA* (aerobactin receptor), and *kpsMT* II (group 2 capsule synthesis) [16]. A similar analysis of 994 avian isolates, all of which were highly pathogenic strains and previously shown to be capable of causing colisepticemia, was used in identifying five genes that could be used as a predictor of an APEC strain, distinguishing it from an avian fecal commensal non-pathogenic *E. coli* strain or AFEC strain [17]. These VF genes, *iutA*, *iroN* (iron metabolism), *iss* (increased serum survival), *hlyF* (hemolysis) and *ompT* (surface exclusion and serum survival) genes were shown to be present on large plasmids, a defining and necessary trait for APEC virulence [10]. Many of these plasmids encode the CoIV bacteriocin, *cvaA, cvaB* (colicin export genes), *traT* (a transfer protein that is involved in serum survival), *tsh* (the temperature-sensitive hemagglutinin gene), *sitA* (iron and manganese transport gene) and *etsAB* (encoding an ABC transport system) [17]. In addition to these plasmid associated genes, isolates were also characterized by the possession of certain chromosomally encoded virulence genes including *fyuA*, (yersiniabactin receptor), the *pap* operon genes (*papA*, *papC*, *papEF*, *papG* that encode parts of the P pilus), capsular biosynthesis genes (K1 and K2 capsule types), and PAI markers (*ibeA*) [18]. 

We recently reported 70% of the *E. coli* isolated from water samples and 60% from the crow fecal samples, obtained from the wetlands of University of Washington Bothell/Cascadia College (UWB/CC) campus (where more than 15,000 crows roost in the autumn and winter months), were resistant to at least one antibiotic [19]. About 40% of these were multidrug resistant (MDR) as they were resistant to ≥three classes of drugs. Extended spectrum beta lactamase (ESBL) containing *E. coli* were isolated from three water (*n* = 184) and nine fecal (*n* = 98) isolates. Six of the fecal and one water isolate belonged to the highly virulent, MDR, and internationally distributed sequence type ST131 by Multi Locus Sequence typing analysis [19]. ST131 is usually a human ExPEC strain. An examination of the phylogroup of these isolates showed that the B2 group was present in 13% and 21% of the *E. coli* isolates from water and feces, respectively, while the D group accounted for another 6.5% of water and 8% of the fecal isolates. This suggested the presence of ExPEC strains in the samples, since the majority of ExPEC isolates have been shown to be associated with *E. coli* phylogenetic groups B2 and D [20].

The aim of the present study was to explore the zoonotic potential of this collection of *E. coli* isolates: by identifying the VF genes that are characteristic of ExPEC strains, and specifically that of APEC sub-pathotype, by checking for plasmid presence, since it has been shown that ExPEC/APEC strains carry large plasmids that encode multiple drug resistance as well as VF genes [18] and by examining the ability of the plasmids to be transferred to other *E. coli* in the environment. Several chromosomal genes that are usually associated with virulence of ExPEC/APEC strains, viz., *fyuA, traT, ibeA, PAI,* cytotoxic necrotizing factor gene (*cnf*), were also examined to further characterize their virulence. An evaluation of the phylogroup associated with the ExPEC strains was also conducted. Presence of IPEC was evaluated by looking for the intimin gene, *eae*, and shigatoxin genes, *stx1* and *stx2* [21].

## 2. Material and Methods

### 2.1. Sample Collections

**Figure 1:**
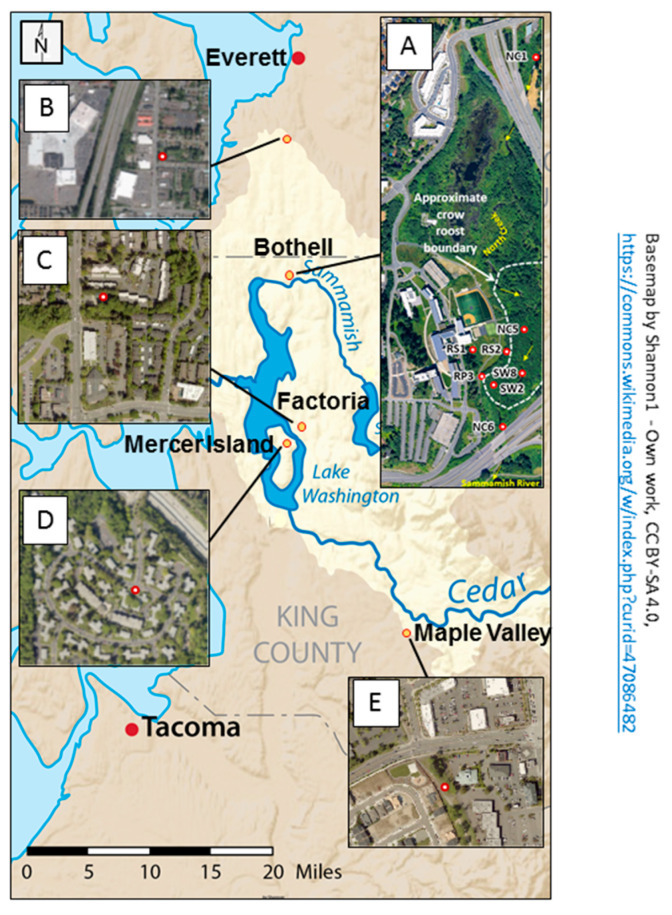
Map showing the locations and characteristics of the sampling areas for the study. The base map indicates that the sampling sites are all in the Lake Washington Watershed (outlined in light tan) of western Washington State. The primary sampling area, displayed in aerial photograph (**A**), is in the 58 acre restored floodplain wetland on the University of Washington Bothell/Cascadia College campus. Red markers indicate locations of surface water collection sites. Site label initials reflect different water characteristics: RS = Runoff Swale; RP = Runoff Pond; SW = Surface Water; NC = North Creek. Water sampled at RS1, RS2, and RP3 flows to these locations via a series of catch basins and pipes from the upland (western) portion of the campus. North Creek originates in Everett and flows south from NC1 to NC6 before discharging into the Sammamish River. Aerial photographs (**B**–**E**) show the urbanized characteristics of surrounding sites (red circles with white centers) where crow fecal samples were collected off campus. (Open source basemap from Shannon1 on Wikimedia. Aerial photograph A from the 2017 UW Bothell Master Plan. Aerial photograph B from Map Everett. Aerial photographs (**C**–**E**) from King County iMap).

Crow feces and water samples were collected simultaneously, during 2014–2015, from the crow roost areas within the UW Bothell/Cascadia College (UWB/CC) campus, and details of the sample collection and isolation of *E. coli* can be found in Sen et al. [19]. Water was collected again from June 2016–April 2017 from the SW8, SW2, NC6, RS1, and RS2 sites before (a day that was preceded by 72 h of no rain) and after rainfall (Figure 1). 

Altogether, 71 fecal isolates and 134 water isolates were analyzed from the wetland area. An additional 34 isolates were collected from non-roost areas, well outside the wetland, such as parking lots and garbage dumps from Factoria (~12 miles away), Mercer Island (~16.5 miles away), Maple Valley (~33 miles away), and Everett (~19 miles away), to estimate how far the impact of the crows extended. Crows were attracted by spreading bread crumbs and were subsequently observed to ensure crow droppings from different birds were obtained.

### 2.2. Isolation of E. coli

Approximately 100 mg of fecal sample was diluted in 500 μL Phosphate Buffered Saline until homogenous suspension was obtained. An aliquot of 10–20 μL of the suspension was directly plated onto Eosin Methylene Blue (EMB) agar and incubated at 37 °C for 24 h. Colonies with metallic green sheen were isolated as putative *E. coli and* further verified as described earlier [19]. Four isolates from each sample were stored at −70 °C in Tryptic Soy Broth (containing 16–20% glycerol until further testing.

Triplicate samples of wetland water were collected in sterile jars. Depending on the turbidity, the samples were diluted either 1:10 or 1:2 with sterile distilled water and a final volume of 100 mL was filtered through 0.45 µm (Hach). *E. coli* and other coliform bacteria colonies were isolated from two of the filters by placing them on m-ColiBlue24 broth (Hach) following the US EPA method 10029 (https://www.hach.com/asset-get.download-en.jsa?id=7639984023, accessed on 14 October 2020). 

The *E. coli* isolated on m-ColiBlue, as blue colonies, were verified on EMB agar, MacConkey agar, and by the presence of the *mdh* gene [19]. Four *E. coli* isolates from each sample were stored at −70 °C until ready for use. The third filter was grown overnight in 5 mL TSY broth. DNA was extracted from 1 mL of this bulk culture, by the rapid boiling method as described below, and was called the “bulk culture DNA”. The remaining 2 mL of the culture was frozen as stock culture after addition of 600 µL of 50% glycerol.

### 2.3. Antibiotic Susceptibility Testing

Colonies grown on Mueller-Hinton agar were used in antibiotic susceptibility testing by the Disk Diffusion method according to Clinical and Laboratory Standards Institute guidelines (CLSI) (CLSI, 2012). The CLSI clinical breakpoints for an antibiotic toward enterobacteriaceae were used to assign isolates sensitive or resistant status. A total of 13 antibiotics were tested: ampicillin (AMP or A) 10 µg, amoxicillin-clavulanic acid (AMC) 20 µg, ceftazidime (CAZ) 30 µg, ceftiofur (XNL) 30 µg, tetracycline (T or TE) 30 µg, ciprofloxacin (CIP) 5 µg, enrofloxacin (ENO) 5 µg, chloramphenicol (C) 30 µg, streptomycin (S) 10 µg, spectinomycin (SPT), sulfamethaoxazole/trimethoprim (SXT), 25 µg, nalidixic acid (NA) 30 µg, and neomycin (N) 5 µg. All antibiotic discs were obtained from Hardy diagnostics. *E. coli* strain, ATCC 35218, was used as quality control strain.

### 2.4. DNA Isolation and PCR

A 1–2 mm size colony from an overnight culture plate was suspended in 50 μL of Prepman Ultra Sample Preparation Reagent (Life Technologies, Foster City, CA, USA). Alternatively, 1 mL of an overnight culture broth of an isolate was centrifuged at 10,000 g for 5 min. The supernatant was removed, and the pellet was resuspended in 200 μL of Prepman Ultra Sample reagent. In either case, the suspensions were heated at 95 °C for 10 min, cooled, and centrifuged at 10,000× *g* for 2 min. Two microliters of the supernatant were directly used in a 20 μL PCR reaction. The supernatants were stored at 4 °C if they were to be used within the week, otherwise they were stored at −20 °C. 

### 2.5. Virulence Gene Detection

The genes that were targeted and detected by PCR were related to (i) bacterial adhesion (*afa/draB, papA, papC, papEF, papG alleles II, sfa/focCD*); (ii) iron acquisition (*fyuA, iroN, iutA*); (iii) serum resistance (*iss, kpsMTII* and *traT*); (iv) toxins and hemolysins (*cnf1, stx1, stx2, hlyA, hlyF*); (v) invasion (*ibeA*). The primers were combined in various pools when used in multiplex assays, as described previously [22] or were used in monoplex assays. For detecting the presence of a gene in a water sample, first the primer pools were tested on “bulk culture DNA”; if a gene was positive by PCR in this sample then individual isolates were tested from that sample. For the APEC pathotype a single multiplex assay was used with primers that targeted the genes *iroN, ompT, iss, hlyF*. Isolates that were positive in the multiplex assay, were verified in monoplex assays and the amplicons obtained further verified by sequencing. This was done because the sizes of the amplicons from *iroN, ompT* and *hlyF* were very similar (Figure S1). Since the *iutA* was tested in the ExPEC assay, it was not tested again here. Either the Jumpstart Reditaq Redi mastermix (Millipore-Sigma) or GemTaq HOTSTART master mix (MGQuest, Lynnwood, WA, USA) was used. The controls used for virulence-associated genes with ExPEC or APEC strains were: ExPEC strain O18:H7 (CDC ID No 00-3038), *E. coli* strain O1:H7 (CDC ID No 00-3166), (O18:H7) CDC ID No 00-3003. These strains were obtained from Dr. Vincent Hill (CDC, Atlanta). For identification of virulence-associated genes typical of Shiga toxin-producing *E. coli* (STEC), ATCC 35150 or ATCC 43889 were used as PCR controls in TaqMan qPCR assays. The genes *stx*_1_, *stx*_2_, *eae,* genes in these strains were detected by a monoplex (*eae*) or duplex qPCR (*stx*_1_, *stx*_2_) as described in [21]. All qPCR reactions were performed in a Mini-opticon icycler (BioRad). For SYBR green PCR, iTaq^TM^ Universal SYBR green mastermix and for TaqMan ^TM^ PCR, iTaq^TM^ Universal Probes Supermix (Bio-rad, Hercules, CA, USA) was used. The cycling parameters for Taqman qPCR were as follows: 1 cycle at 95 °C for 10 min, followed by 40 cycles of 15 s at 95 °C, 30 s at 58 °C, and 30 s at 72 °C, with a final cycle of 5 min at 72 °C. Each isolate was tested for a gene at least twice.

### 2.6. Detection of Antibiotic Resistance Genes and Plasmid Incompatibility (INC) Sites

For determining the presence of plasmid/s in an isolate, polymerase chain-based replicon typing was used. The method of Johnson et al. [23] was employed, where 18 plasmid replicons are identified by using 3 multiplex PCR assays. Total genomic DNA from the isolates was obtained by the boiling method as described above. An additional monoplex that targeted only the Frep inc gene was used. The cycling parameters for the monoplex was the same as the multiplex reactions except that an annealing temperature of 55 °C was used [24].

For detecting the presence of antibiotic resistance genes, *bla*_cmy-2,_ *bla*_ctx-M,_ *tet*(A), *tet*(B), *tet*(M), *str*A, and *str*B in the isolates as well as the trans-conjugants, SYBR green, or TaqMan based qPCR was used. The methods have been described earlier [19]. 

### 2.7. Mating Experiments

The mating was done as described previously [25]. Donor strains were grown on LB (Luria-Bertani) plates containing antibiotics (as per donor’s antibiotic resistance listed in Table 2), from frozen stocks, by overnight incubation at 37 °C. The antibiotics on the plate were used at concentrations of ampicillin (32 μg mL^−1^), tetracycline (16 μg mL^−1^), streptomycin (16 μg mL^−1^), ceftiofur (8 μg mL^−1^), and Cefotaxime (2 μg mL^−1^). Thus, F46.2 was grown on LB + Amp + Str. All donors were tested for their inability to grow on LB + nalidixic acid (NA) (32 μg mL^−1^). The recipient strain, *E. coli* K-12^NA^, was obtained from Dr. Douglas Call (Washington State University, WA, USA). It was grown on LB + NA (32 μg mL^−1^). The day before mating, donor and recipient strains were grown separately in LB Broth by overnight incubation at 37 °C. Equal quantities (10 μL) of overnight cultures of donor and recipient strains were placed on top of a nitrocellulose membrane that was overlaid on LB agar with no antibiotics. After 24 h of incubation at 37 °C, cells were suspended from the membrane in 500 μL of sterile phosphate-buffered saline (PBS, pH 7.0) and spread onto LB agar plates containing 32 μg mL^−1^ nalidixic acid and at least one other antibiotic to which the donor cells were resistant. Colonies that grew on these selective agar plates were considered trans-conjugants.

Trans-conjugants were screened for their donor’s antibiotic resistance genotypes, such as the presence of *strB, tet*(A), *tet*(B), *bla*_TEM-1_, *bla*_SHV_, *bla*_CMY_, and *bla*_CTX-M_ genes as described previously [19]. 

### 2.8. Phylogenetic Studies

Most of the fecal isolates were typed in the earlier described study [19]. Fecal and water isolates from Round 6 (June 5, 2016), and water isolates obtained from June 22, 2016 to January 31, 2017 were phylogenetically typed in this study. The quadruplex PCR method of Clermont et al. was used to assign the *E. coli* isolates to one of the eight phylogenetic groups [26]. After initial placement into four groups, based on the results of the quadruplex, strains belonging to phylo-groups A and C or D and E were further identified by using C and E specific primers. [26].

### 2.9. Statistical Analysis

Fisher’s exact test was used to identify significant differences between occurrence data, which is represented as percentage, such as percent of a virulence factor gene present in fecal isolates versus those among water isolates. The *p* values corresponding to the differences between 2 groups are reported in the tables below the graphs. *p* values corresponding to <0.05 were considered significant.

## 3. Results

### 3.1. Relative Abundance of Virulence Genes in Crow Feces and Water Isolates

**Figure 2:**
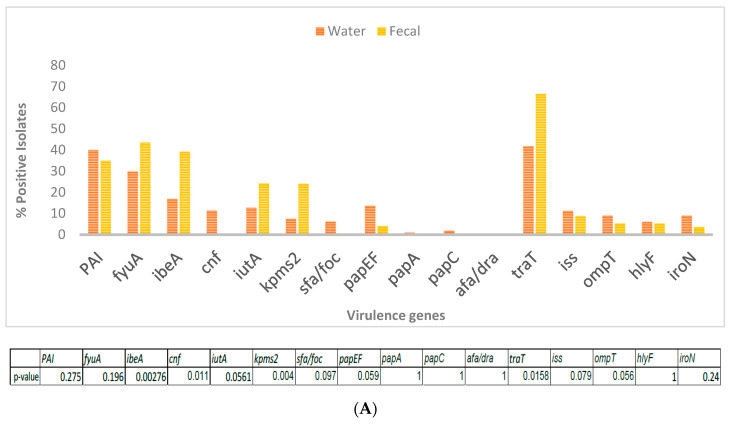

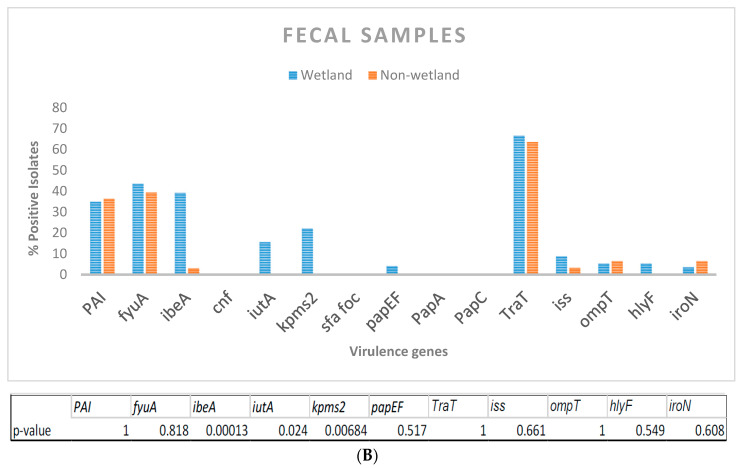
(**A**) Presence of virulence genes in *E. coli* isolated from wetland water (*n* = 134) and crow feces (*n* = 71) was determined by 4 multiplex PCRs with primers targeted to 15 genes. Table indicates significant difference in virulence gene abundance between water and fecal isolates. (**B**) Percentage of virulence genes in wetland fecal isolates (*n* = 71) compared to that present in isolates from 3 non-wetland locations (*n* = 34). *p*-values are indicated below.

The fecal *E. coli* isolates from 2014–2015, were compared with *E. coli* water isolates from the same period for the presence of 13 virulence factor genes. DNA isolated from bulk cultures from each water sample (e.g., SW 2) were first tested for the presence of a gene before testing for 2 individual isolates from that site (e.g., SW 2.1, SW 2.2 or RS 1.1, RS 1.3). All genes tested were detected in DNA from bulk cultures. The virulence genes *cnf* and sfa/foc were absent in the fecal isolates, while afa/dra was completely absent in all water and fecal isolates (Figure 2A). Among the genes that were present in both fecal and water isolates, *traT* and *fyuA* were the most abundant. There was significant difference in the presence of *ibeA* (*p* = 0.0028), *kpsMT-II* (*p* = 0.004) and *traT* (*p* = 0.016) between the 2 types of isolates, with all three being present in greater numbers among the fecal isolates.

When fecal isolates from crows within and outside the UWB/CC wetland area were compared, *traT* and *fyuA* were again the most abundant genes from all areas. *ibeA, iutA* and *kpsMT-ll*, genes were significantly more abundant in the wetland fecal isolates (Figure 2B). 

### 3.2. Identification of EXPEC, IPEC, STEC Strains

For identifying potentially ExPEC strains based on the presence of ≥2 of the 5 cardinal virulence factor genes, the water and fecal isolate collection from 2014–2015, as well as the water isolate collection from 2016–2017 that included stormwater samples, were evaluated as described earlier. In total, 10 of 71 fecal isolates (14%) and 15 of 134 water isolates (11.2%) were potential ExPEC strains.

ExPEC-like strains (Table 1). Stormwater events (rain days) did not appear to contribute more than usual pathogenic-like *E. coli* (Table 1), with 1 isolate from 10/4/16 and 7 isolates from 1/31/17. Although the *eae* gene was detected in a couple of bulk culture samples, no *stx* 1 or *stx* 2 genes were identified, and therefore IPEC of the enteropathogenic *E. coli* (EPEC) pathotype and Shiga toxin-producing *E. coli* (STEC) were considered absent [7]. 

### 3.3. Identification of APEC Strains and Phylogenetic Types of the Isolates 

The isolates that showed the presence of *iutA* in the previous multiplex assays, for determining the ExpEC strains, were further analyzed for the presence of *iss, hlyF, ompT,* and *iroN* genes for identifying the potential APEC-like strains. This strategy allowed us to definitively identify the presence of the *iss* gene with an amplicon size of 323 bp, distinguishing it from *iutA,* 302 bp. In total, 14 of 134 water isolates and 6 of 71 fecal isolates could be characterized as APEC, respectively (Table 1). Among the 20 APEC-like strains, 12 (60%) were identified as B2 phylotype, while 24 of all 37 characterized either as ExPEC or APEC were of the B2 phylotype (64.9%).

### 3.4. Comparison of Antibiotic Resistance of Fecal Isolates from Wetland and Non-Wetland Areas

**Figure 3:**
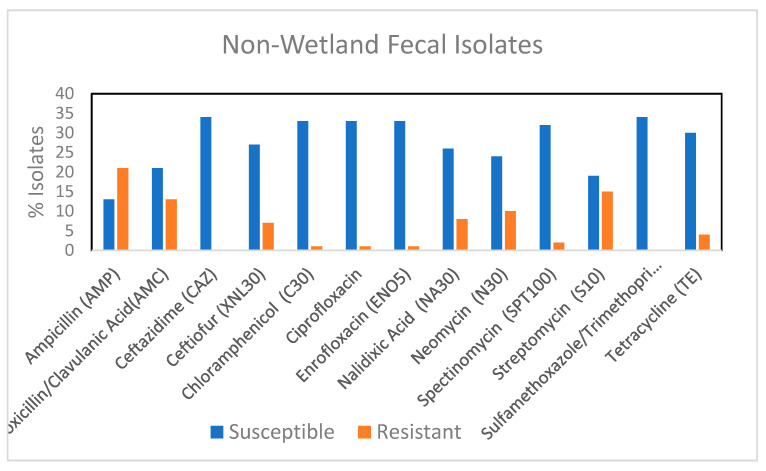
Percentage of fecal *E. coli* isolates from non-wetland (*n* = 34) showing resistance and susceptibility to 13 selected antimicrobials. AMC, amoxicillin/clavulanic acid; AMP, ampicillin; XNL, ceftiofur; C, chloramphenicol; CAZ, Ceftazidime CIP, ciprofloxacin; ENO, Enrofloxacin NA, Nalidixic acid, N, Neomycin, STR, streptomycin; SPT, spectinomycin, TE, tetracycline; SXT, trimethoprim/sulfamethoxazole.

None of the non-wetland isolates were resistant to ceftazidime, which indicated a potential lack of ESBL containing strains (Figure 3). When these fecal isolates were compared with the wetland fecal isolates, other than to ceftazidime (*p* < 0.00072) there were no differences in the proportion of isolates that were resistant to an antibiotic between the two groups (Figure S2). 76.4% of the non-wetland isolates were resistant to at least one antibiotic, while 17.6% were MDR In contrast, wetland fecal isolates had 71.4% isolates resistant to one antibiotic and 35.7% were MDR. Since no ExPEC strain was found among the non-wetland isolates, it suggests the presence of ExPEC strains may change a population’s MDR profile. Water can further impact the number of such strains, by allowing for their spread.

### 3.5. Replicon Typing and Transferability of Plasmid

Altogether, 59 isolates were subjected to polymerase chain-based replicon typing. The isolates were selected if they were resistant to 2 or more classes of antibiotics or displayed a virulence genotype that suggested the presence of plasmid-borne genes. FIB was the most abundant replicon type found, followed by FI1 (Table 2). Several strains had multiple replicon types. When subjected to mating, 33 of 38 strains (17 of 18 fecal and 16 of 20 water isolates) tested were successfully mated. Selection was performed on different combinations of antibiotics, all of which contained NA (recipient’s resistance marker). The trans-conjugants, when tested for the respective donor’s AR genes, usually showed their presence (Table 3, Figure S2). In six of the eight strains tested, the virulence genes associated with APEC-like strains were transferred (Table 3, Figure S3), which is not surprising because virulence and R plasmids have been shown to co-transfer during conjugation in APEC isolates [23].

The six ESBL containing, ST131 isolates transferred the AR profile (Figure S2), but not the virulence profile, e.g., *iutA, traT, iss*.

## 4. Discussion

The combination of virulence genes and the phylogenetic background, rather than the possession of one or more virulence genes or the ecological background, has been a useful predictor of the ability of an ExPEC strain to cause disease, at least in mouse models and chicks [17,27,28]. A set of five virulence genes commonly found in ExPEC [16] and another set typically found on a colicin encoding plasmid in APEC strains [17] were examined in 104 crow isolates and 134 wetland water isolates. Strains possessing one of these five genes were rejected as potentially APEC, because a screening of 994 avian isolates had earlier demonstrated that non-pathogenic Avian fecal *E. coli* or AFEC strains had on the average 1.3 of these virulence genes while APEC strains had three or more [17]. Of the 20 APEC-like (6 fecal and 14 water) strains that were classified in this study: 18 strains harbored the Inc F1B replicon, 7 had more than one replicon, and 12 carried a colicin plasmid, all lending additional support to these strains being of APEC-like pathotype as reported by Johnson et al. [23]. As noted earlier, several studies have pointed out that ExPEC and APEC strains share a common virulence factor gene pool that is flexible, and all stages of infection can be modulated by alternative virulence factors in a “mix and match combinatorial” manner [27,29]. Thus, our analysis of the set of five genes may not be fully representative of the true picture. Furthermore, chromosomal virulence factor genes could compensate for the absence of the typical genes found on a plasmid and thus several strains rich in chromosomally located virulence genes may be proven to be APEC (e.g., F15.2 and F42.2). Undoubtedly, animal challenge studies would provide more definitive results. This study provides a baseline on which such studies can be based, serving as a screening tool for an unknown group of isolates.

A screening of the overall virulence gene profile of the fecal isolates (wetland and greater Seattle area) revealed that *fyuA,* an iron metabolism gene was present in about 40% of isolates, with it being over represented in ExPEC/APEC-like fecal strains (66.7% occurrence). It has been speculated that the presence of several iron metabolism genes, in this case *fyuA, iroN,* and *iutA*, may be important in establishing avian colibacillosis, iron acquisition being critical for a pathogen to establish infection [10]. Similarly, the hemin receptor *chuA,* which has been shown to have a significant association with the serum resistance phenotype in APEC virulence [27], was present in 73% of the ExPEC/APEC strains and overall, more in APEC than among AFEC strains. *traT* that encodes a transfer protein and provides complement resistance, was present in about 65% of all fecal isolates including the AFEC isolates from the greater Seattle area. This suggests that although this gene product may be necessary for serum bactericidal activity, it may not be sufficient, and the repertoire of other virulence genes products that characterize most APEC strains are needed for further establishment of infection. A couple of other studies have reported *traT* to occur in commensal AFEC as well as APEC strains with equal abundance [10,27], and suggested that either *traT* was not a useful marker for in vivo virulence of APEC strains, or that fecal strains can act as a reservoir for extraintestinal infection causing genes [30]. Since *ibeA*, a recognized NMEC virulence factor, is involved in biofilm formation and avian fibroblast invasion [31], its presence in greater numbers in the fecal isolates suggests that the crow gut, like the chicken gut, may support ExPEC *E. coli* [27]. Further research is required to determine whether those strains harboring a higher number of virulence factor genes (e.g., F11.2, F13.2, F46.2) have higher pathogenicity or are more virulent in in vivo studies.

Since environmental patterns are imposed on the indigenous population structure of *E. coli* after fecal deposition [32], the finding of virulence gene presence in the wetland water similar to that of crow isolates is not unexpected. However, certain genes such as *sfa/foc, cnf, papEF* were only present in the water samples, while others such as *traT* and *ibeA* occurred in significantly less proportion relative to fecal isolates. The VF genes seen only in the water isolates could be from *E. coli* strains that resulted from the overflow of the North Creek and/or washings from neighboring sites that border the roost areas, into the wetland, such as RS1, RS2, into SW8, and SW2 sites. Since the wetland water is influenced by the North Creek that flows past it, it is possible that besides feces, there may be other contributors of *E. coli* that do not contain *traT* or *ibeA*. In water isolates there was a representation of the *pap* genes, especially *papEF* (13%) which was present in less than 3% of the fecal isolates which is very similar to that seen in chickens [10]. Water isolates had a slightly greater percentage of APEC-like (9.7% vs. 4%) isolates but overall, the total percentage of ExPEC/APEC were about the same (15.6% and 17.5%). However, our analysis is based only on the presence of the five cardinal genes. Other virulence factor genes, including the chromosomally located ones, will have a role in determining the overall virulence. Thus, the numbers of ExPEC- and APEC-like isolates detected must be treated with caution.

In order to see the role of the wetland in dissemination of virulence, isolates from non-wetland areas were explored. Importantly, no ExPEC was found in isolates from the non-wetland sites. However, the sample size from each of the four locations was small, and perhaps this finding is not representative. Alternately, the wetland water which the crows drink may contribute to the spread of pathogenic isolates in the wetland area as the crows defecate in the same area. Except for Round 5 where sufficient water isolates could not be tested, ExPEC/APEC-like isolates were found in all rounds in both feces and water.

A large percentage of the fecal ExPEC/APEC (73%) were MDR. Other studies have reported such positive correlation between virulence and presence of antimicrobial resistance in Avian *E. coli* isolates [20,33], and indicated them to be reservoirs for MDR of human ExPEC, as well as the commensal *E. coli* populations. We had earlier reported that overall 35.5% strains of the fecal isolates from the wetland were MDR [19]. Most of the drug resistant strains’ genes were carried on plasmids as demonstrated in our mating studies. Thus, AmpC beta lactamase due to presence of *bla*_CMY-2_ found in both water and fecal isolates could be transferred from all but one of the donor strains, to a recipient strain lacking this gene. *E. coli* possessing *bla*_CMY-2_, a gene that was originally derived from *Citrobacter* spp._,_ is the most common plasmid-mediated cephamycinase reported in *E. coli* strains from hospitals all over the world [20], including the U.S. [34,35]. Importantly, ExPEC strains are being increasingly implicated in possession of these and other ESBL genes [20]. Our results provide evidence that large corvids serve as vectors for the transmission of such antibiotic resistance determinants. Other genes that could be transferred were the *bla*_ctx-M,_ *tet*(A), *tet*(B) genes, *str*A, and *str*B genes. Genes encoding multiple resistance are usually found on transmissible R plasmids, and multidrug-resistant APEC strains carrying such large conjugative plasmids have been demonstrated in several studies [18,36,37]. The virulence genes and AR determinants were reported to be co-transferred to a recipient strain in these studies. In our study *colV* plasmid could be transferred in six isolates, two of which were also MDR. Although it was shown that the transfer of APEC plasmid to a commensal *E. coli* resulted in its killing chicken embryos and causing UTI in mice [38], the mobilization of virulence genes from a virulent strain to an avirulent one may not always result in the recipient becoming a pathogen [36]. Again, further in vivo studies are warranted to understand the true pathogenic nature of the trans-conjugants.

The main phylogenetic group associated with the ExPEC/APEC pathotype in this study was the EcoR B2 group, a result most frequently reported [27,39,40]. Furthermore, it was recently shown that intestinal strains of the EcoR group B2, that also had the ExPEC virulence factors, correlated well with successful colonization of piglets [41]. Thus, this result of ExPEC strains found in the gut of the crow is not unexpected. Our determination of sequence types (ST) of this collection of isolates in the earlier study [19], show several of the isolates characterized as ExPEC by the VF gene method reported here, belong to ST 131, ST58, ST 83, ST357. These STs have been earlier isolated from birds including crows, poultry, companion animals, livestock, water, as well as humans [42,43], and were shown to be APEC and ExPEC strains, as also non-pathogens in the enterobase database (http://enterobase.warwick.ac.uk/species/ecoli/search_strains?query=st_search, accessed on 14 October 2020). Since the wetlands are very close to the nearby Snohomish County, WA, where there is abundant agricultural and rural land, it is possible that the crows acquired some of these strains from the farm animals that live there. This indicates crows can act as vectors in the transmission of pathogenic and drug resistant *E. coli*; they may also be transmitting some of the *E. coli* they acquired during their roosting to other animals during their daytime activities.

Our determination of the combination of virulence genes present and the phylogenetic type show that crows bring in pathogenic *E. coli.* Some of them can be potentially characterized as APEC-like, others ExPEC, and some that can potentially be both ExPEC and APEC sub-pathotypes, which is not surprising since APEC is a sub-pathotype of ExPEC. Several strains are MDR and the resistance plasmids are mobile. The wetland allows for their further dissemination, probably through the corvids’ interactions with surface water during their daily visitation to the wetland and subsequent day time scavenging activities to even farther places. Since they are also partially migratory, with populations moving towards southern latitudes of North America during the winter, these strains may be carried farther during these months, implicating the crow even more in the spread of zoonosis [44]. However, the strains themselves do not persist in the water for long, because in the earlier study we showed that the same sequence types could not be recovered in subsequent collections [19]. Future studies will be directed to understanding the survivability of these strains in the wetland.

## Figures and Tables

**Table 1:** Virulence Profile of Fecal and Water Isolates.

Sample	*PAI*	*fyuA*	*ibeA*	*cnf*	*iutA*	*kpmsT II/KPs1*	*Sfa/foc*	*papEF*	*papA*	*papC*	*traT*	*iss*	*ompT*	*hlyF*	*iroN*	*chuA*	*cva*	Potential Pathotype	Phylotype	Replicon Type
**FECAL**																				
F11.1 (R2)	1	1	0	0	1	1	0	1	0	0	1	0	0	0	0	1	0	ExPEC	B2	FIB
F11.2 (R2)	1	1	0	0	1	1	0	0	0	0	1	0	0	0	0	1	0	ExPEC	B2	FIB
F13.1 (R2)	1	1	0	0	1	1	0	1	0	0	1	0	0	0	0	1	0	ExPEC	B2	FIB
F13.2 (R2)	1	1	0	0	1	1	0	1	0	0	1	0	0	0	0	1	0	ExPEC	B2	FIB
F15.2 (R2)	1	1	1	0	1	1	0	0	0	0	1	1	0	0	0	1	0	ExPEC	B2	FIB
F16.2 (R2)	1	1	1	0	1	1	0	0	0	0	1	0	0	0	0	1	0	ExPEC	B2	FIB
F39.2 (R3)	0	0	1	0	1	1	0	0	0	0	1	0	0	0	0	0	0	ExPEC	B1	FIC
F42.2 (R4)	1	1	1	0	1	1	0	0	0	0	1	0	1	0	0	0	0	ExPEC	B1	B/O
F43.1 (R4)	0	1	1	0	1	0	1	0	0	1	1	0	0	0	0	0	0	ExPEC	A	B/O, A/C
F46.2 (R4)	0	0	0	0	0	1	1	0	0	0	1	1	1	1	1	1	1	ExPEC/APEC	B2	FIB
F47.1 (R4)	0	0	0	0	1	1	0	0	0	0	1	1	0	0	1	0	0	APEC	C	I1, FIB
F53.1 (R5)	0	0	0	0	1	1	0	0	0	0	1	1	0	1	1	0	0	APEC	B1	I1
FS 7 (R6)	0	0	0	0	1	0	0	0	0	0	1	1	1	1	1	1	1	APEC	cryptic clade	FIB
FS1 (R6)	0	0	0	0	1	0	0	0	0	0	1	1	0	0	1	1	1	APEC	F	FIB
FS4 (R6)	0	0	1	0	1	0	0	0	0	0	1	0	0	0	1	1	1	APEC	F	FIB
**WATER**																				
NC5.1ctx (R2)	1	1	0	0	1	1	0	1	0	0	1	0	0	0	0	1	0	ExPEC	B2	FIB, Frep
NC6.4ctx (R2)	0	0	0	0	1	0	0	0	0	0	1	1	1	1	1	0	1	APEC	B1	FIB
SW2.1 (R3)	0	0	1	0	1	0	0	1	1	1	0	1	0	1	1	1	1	ExPEC/APEC	B2	FIB, I1
RP3.2 (R4)	0	0	1	0	0	1	1	1	1	0	0	1	1	0	1	0	0	ExPEC/APEC	B1	B/0; I1
SW2.26/22/16 (No Rain)	0	1	0	0	1	0	0	0	0	0	1	1	1	1	0	1	1	APEC	B2	I1, FIB, FIA
RS1.1 6/22/16 (No Rain)	1	0	0	0	0	0	1	0	1	0	0	0	0	0	0	1	0	ExPEC	B2	FIB
RS1.3 6/22/16 (No Rain)	1	0	0	1	1	1	0	0	1	0	1	0	1	1	1	1	0	EXPEC/APEC	B2	FIB
RS2.2 6/22/16 (No Rain)	1	1	1	0	1	0	0	0	0	0	0	1	1	1	0	1	1	APEC	B2	FIB
NC6.1 6/22/16 (No Rain)	0	1	1	0	1	0	0	0	0	0	1	1	1	1	0	1	1	APEC	B2	FIB, FIA
NC6.2 6/22/16 (No Rain)	1	1	1	0	1	1	0	0	0	0	0	1	0	1	0	1	1	ExPEC/APEC	B2	FIB, FIA
NC1.1 7/9/16 (No Rain)	1	1	1	0	0	1	1	1	0	0	0	0	0	0	1	1	0	ExPEC	B2	No ID
NC1.2 10/3/16 (No rain)	1	1	0	1	0	1	0	0	0	0	1	0	1	0	1	1	0	APEC	B2	FIB
NC1.3 10/3/16 (No rain)	1	1	1	0	1	0	1	1	1	1	1	0	0	0	0	1	0	ExPEC	B2	FIB
RS2.3 10/3/16 (No Rain)	1	1	1	0	1	0	1	1	1	1	1	0	0	0	0	1	0	ExPEC	B2	FIB
SW8.1 10/4/16 (Rain)	0	1	0	0	1	0	0	0	0	0	1	1	0	0	1	0	0	APEC	B1	FIB
RS1.1 1/31/17 (Rain)	1	1	0	0	1	0	0	1	0	0	1	0	0	0	0	0	0	ExPEC	A	I1
NC6.1 1/31/17 (Rain)	0	1	1	0	1	0	1	1	0	0	0	1	1	1	0	1	0	ExPEC/APEC	B2	FIB, FIA
RS2.2 1/31/17 (Rain)	1	1	0	0	1	0	0	1	0	0	1	1	1	1	1	1	1	APEC	B2	FIB
NC1.2 1/31/17 (Rain)	1	1	1	0	0	1	1	1	0	0	1	ND	ND	ND	ND	1	0	ExPEC	B2	No ID
RS2.3 1/31/17 (Rain)	1	1	0	0	1	0	0	1	0	0	1	1	1	1	1	1	1	ExPEC/APEC	B2	FIB
NC1.3 1/31/17 (Rain)	1	1	0	0	0	0	1	1	0	0	1	1	ND	ND	ND	0	-	ExPEC	B1	FIB
SW2.21/31/17 (Rain)	1	1	1	1	0	0	0	0	1	1	1	0	1	1	0	1	0	ExPEC/APEC	B2	FIB

The virulence profile of the potential ExPEC and APEC-like strains are presented. The phylogenetic group and the plasmid incompatibility types are included for the strains. Different rounds of collection where both feces and water samples were collected, are denoted as: 8-20-14 (R1) 9-5-14 (R2), 1-21-15 (R3), 2-27-15, (R4), 4-5-15 (R5), 6/5/16 (R6). Rain day samples, when only isolates from water were collected, are on 10/4/16 and 1/31/17 while no rain days are 6/22/16 (R6), 7/9/16, 10/3/16. All fecal isolates (denoted by F) were obtained from the roost area outlined by white dashes in the map A (Figure 1). The water isolates are indicated by the prefixes of sites from where they were collected and the initial details can be found in the legend under Figure 1. Thus, NC5, SW2, and SW8 are sites within the roost area, while RS2 and RP3 are right at the margin of the roost area. NC6 is just downstream of the roost area, while NC1 and RS1 are sites farther away from the actual roost area (map A, Figure 1).

**Table 2:** Distribution of Replicon types among 27 water and 32 Fecal isolates from the Wetland.

Replicon Type	Number of Isolates	%
FIB	35	59.32
I1	15	25.42
FIC	3	5.08
F1A	8	13.56
P	1	1.69
B/0	7	11.86
FIIA	1	1.69
FIB, I1, F1C	1	1.69
F1A, FIB	5	8.47
F11A, FIB, B/O	1	1.69
I1, FIB, IA	1	1.69
FIB, B/O	2	3.39
A/C	1	1.69
P, FIC	1	1.69

**Table 3:** Antibiotic Resistance Pattern of Donors and Trans-conjugants.

Strain ID	Resistance Phenotype *	AR Genes *	Mating	Replicon Type	Trans Conjugant
**Fecal**		**Donor**		**Donor**	
F11.1	AMP-CTX-CF-XNL-S-SXT-T	*bla*_ctx_, *str*A, *sul1*, *tet*(A), *tet*(M)	1	FIB	*bla_ctx-M_*, *str*B
F11.2	AMP-CAZ-CF-XNL-S-SXT-T	*bla*_ctx_, *str*A, *str*B, *sul1*, *tet*(A), *tet*(B), *tet*(M)	1	FIB	*bla ctx-M*, *- tetA*, *str*B
F13.1	AMP-CAZ-CF-XNL-NA-S-SPT-SXT-T	*bla*_ctx_, *str*A, *str*B, *sul1*, *tet*(A)	1	FIB	*bla ctx-M*, *str*B
F15.2	AMP-CAZ-XNL-N--S-SXT-SPT-T	*bla*_ctx-M_, *str*A, *str*B, *sul1*, *tet*(A), *tet*(M), *tetB*	1	FIB,	*bla ctx-M*
F16.2	AMP-CAZ-CF-XNL-NA-SXT-T	*bla*_ctx-M_, *bla*_CMY-2_, *str*A, *str*B, *sul1*, *tet*(A)	1	FIB	*bla ctx*, *str*B
F35.2 ctx	AMP-CAZ	*bla_CMY2_*, *strB,*	1	I1	*bla_CMY-2_*
F 42.2	AMP-AMC-CAZ-XNL-NA-N-S	*bla*_CMY-2_, *str*B	1	B/O	*bla-_CMY 2_*, *str*B
F43.1	AMP-AMC-XNL-C-NA-N-S-T	*str*B, *tet*(B), *tet*(M)	0	B/O, A/C	*-*
F46.2	AMP-CAZ-NA-N-S-T	*bla*_CMY-2_, *str*B, *tet*(B)	1	FIB	FIB, *iss*, *iutA, iroN*
F47.2	AMP-AMC-XNL-ENO-NA-N-S	*bla*_tem_, *str*B, *IS133*	1	FIB, I1,	*str*B
F39.2	AMP, S, CIP, CAZ, T	*bla_CMY-2_*, *tetA, strA*	1	F1C, P	*bla_CMY-2_*
F53.1	AMP	*bla_CMY2_*, *str*B	1	I1	I1, *bla_CMY-2_*, *str*B
FS1	AMP, N, T	*bla_CMY2_*, *tet* (A)	ND	FIB	-
FS7	AMP, T	*bla_CMY2_*	1	FIB	*bla_CMY2_*, *cvaC*
FS4	AMP, T	*bla_CMY2_*	ND	FIB	-
**Water**					
NC5.1ctx 9/17/14	AMP-CAZ-XNL-S-SXT--T	*bla*_ctx_, *str*B, *sul1*, *tet*(A), *tet*(M)	1	FIB, Frep	*bla ctx-M*, *str*B
NC6.4ctx 9/17/14	T	*tet*(A)	1	FIB, FIC	*tet*(A), *iroN*, *iss*, *iutA*
SW2.1 1/21/15	Amp-NA-S	*bla_CMY-2_*, *str*B	1	FIB, FI1	*bla_CMY-2_*
RP3.2 2/27/15	AMP-AMC-CTX-XNL-ENO-NA-N	*bla*_CMY-2_	0	I1, B/O	*-*
RS 1.1 6/22/16	AMP-S-T	*bla_CMY-2_*, *tet(*A), *str*A	1	FIB	*bla_CMY-2_*
RS1.3 6/22/16	AMP-CAZ-CF-XNL-S-SXT-C	*bla_(CTX-M)_*, *tet*(B), *str*A	0	FIB	*str*A
SW2.2 6/22/16	T (tet)	*tet*(A)	ND	I1, FIB, FIA	ND
RS2.2 6/22/16	AMP-S-T	*str*A, *tet*(M), *tet*(A)	1	FIB,	*tet*(A), *ompT*, *hlyF*
NC6.1 6/22/16	AMP-T	*bla_CMY-2_*, *tet*(A)	1	FIB, FIA	*tet*(A), *iss*, *ompT*, *hlyF*
NC6.2 6/22/16	AMP-T	bla_ctx_, *tet*(A)	1	FIB, FIA	*tet*(A), *iss*, *ompT*, *hlyF*
NC1.1 7/9/16	AMP, S	*bla_CMY-2_*	1	No ID	*bla_CMY-2_*
NC1.2 10/3/16	S	*str*B	0	FIB	-
NC1.3 1 10/3/16	Amp-S-SXT	*bla_CMY-2_*	1	No ID	*bla_CMY2_*
RS2.3 10/3/16	S	*str*B	ND	FIB	ND
SW8.1 10/4/16	AMP-S-SXT	*str*A	ND	FIB	ND
RS1.1 1/31/17	SXT-T	*tet*(A), *tet*(D)	ND	I1	ND
NC6.1 1/31/17	T (tet)	*tet*(A)	1	FIB, FIA	*tet*(A)
RS2.2 1/31/17	AMP-CTX-S, T	*tet*(A), tet(B), aadA strB	1	FIB	*tet*(A), *tet*(B)
NC1.2 1/31/17	AMP-T	*bla-*_CMY-2_, *tet*(M)	1	ND	*bla*_CMY-2_
RS2.3 1/31/17	AMP-S-SXT-T	*str*B, *aad*E, *tet*(B)	1	FIB	*tet*(B)
NC1.3 1/31/17	AMP-S-SXT-T	*bla*_CMY-2_, *tet*(B)	1	FIB	*bla*_CMY-2_, *tet*(B)
SW2.2 1-31-17	SXT-T	*tet*(A), *tet*(D)	0	FIB	-

0 indicates no mating and 1 successful mating. ND is not determined. The trans-conjugants were characterized by presence of one or more AR gene and/or virulence gene, present in the donor. The dates of collection of the fecal isolates are presented under the legend to Table 1, * Some of the data in these columns were reported in Sen et al. [19]. Data of 1/31/17 (water samples) and FS1, FS7 and FS3 fecal samples, were obtained in this study.

## Supplementary Materials

The following are available online at https://www.mdpi.com/2076-2607/8/10/1595/s1, Figure S1: PCR products of quadruplex performed with primers for iroN, hlyA, OmpT and iss on water and fecal (F) isolates. Samples were run on 2 % gel. Figure S2. Percentage of fecal E. coli isolates from non-wetland (*n* = 34) and wetland (*n* = 71), showing antimicrobial resistance to 13 selected antimicrobials. Figure S3: PCR products for bla-CMY-2, 454 bp (A) or bla-ctx-M, 554 bp (B), obtained from donor (D) ExPEC/APEC strains and corresponding trans-conjugants(Tc) following mating with E. coli K12.
