# Supplementary material for: American Crows as Carriers of Extra Intestinal Pathogenic E. coli and Avian Pathogenic-Like E. coli and Their Potential Impact on a Constructed Wetland
Source: Microorganisms. 2020 Oct 16;8(10):1595. doi: 10.3390/microorganisms8101595 (PMC7602749; doi:10.3390/microorganisms8101595)
Supplement: Supplementary file 1 [file microorganisms-08-01595-s001.pdf]

Figure S1

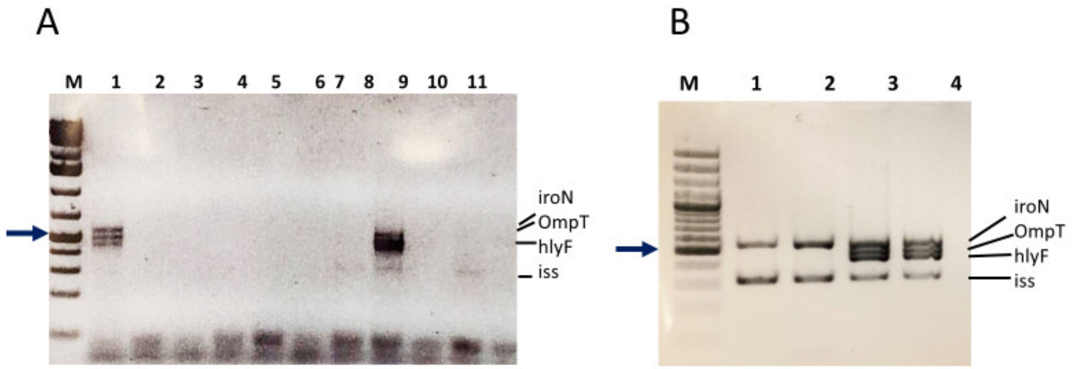

Figure S1. PCR products of quadruplex performed with primers for *iroN, hlyA, OmpT and iss* on water and fecal (F) isolates. Samples were run on 2 % gel **A**. Lane 1-11 Products from positive control (PC), NC1.2 (10/4/16), SW2.4 (5/16), RS 2.2 (10/4/16), RS2.3 (10/4/16), F41.1 (2/15), RS1.1 (1/17), RS2.3 (1/17). SW8.3 (7/16), F35.2 (2/15), F20.3 (1/15), respectively. Water isolates are indicated by the sites: SW2, SW8, RS or NC they were isolated from, and dates of isolation (indicated in brackets) **B.** PCR products of quadruplex for water isolate NC 6.4_{ctx} (9/14 or R2) (lane1) and F46.2 (lane 4), and from transconjugants obtained from mating NC 6.4_{ctx} (9/14) (lane2) and F46.2 (lane 4) with *E. coli* K12 ^{NA}, respectively. M is the GeneRuler 100 bp Plus DNA ladder (Thermoscientific) with the arrow pointing to 500 bp.

Figure S2

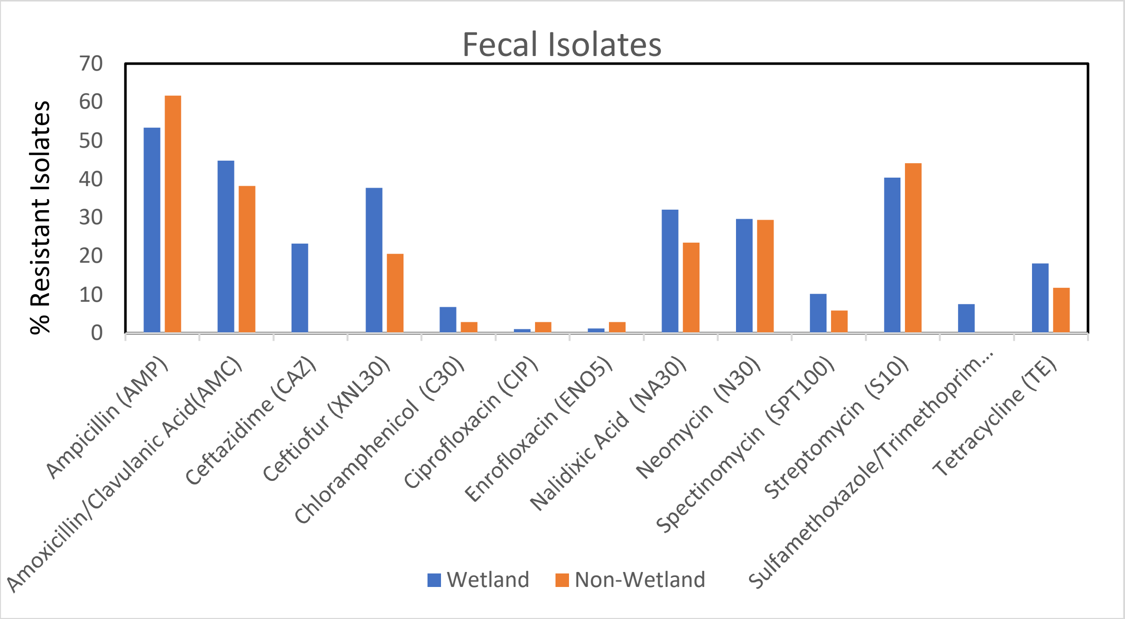

	AMP	AMC	CAZ	XNL 30	C30	CIP	ENO5	NA30	N30	SPT100	S10	SXT	TE
p-value	0.545	0.685	0.00072	0.061	0.664	0.468	0.502	0.508	1	0.721	0.684	0.104	0.586

Figure S2. Percentage of fecal *E. coli* isolates from non-wetland (n = 34) and wetland (n = 71), showing antimicrobial resistance to 13 selected antimicrobials. Table indicates significant differences in antibiotic resistance between fecal isolates of the 2 groups for an antibiotic according to Fisher's Exact test. The antibiotic resistance data of wetland isolates (indicated in blue) was taken from the previous study (19)

Figure S3

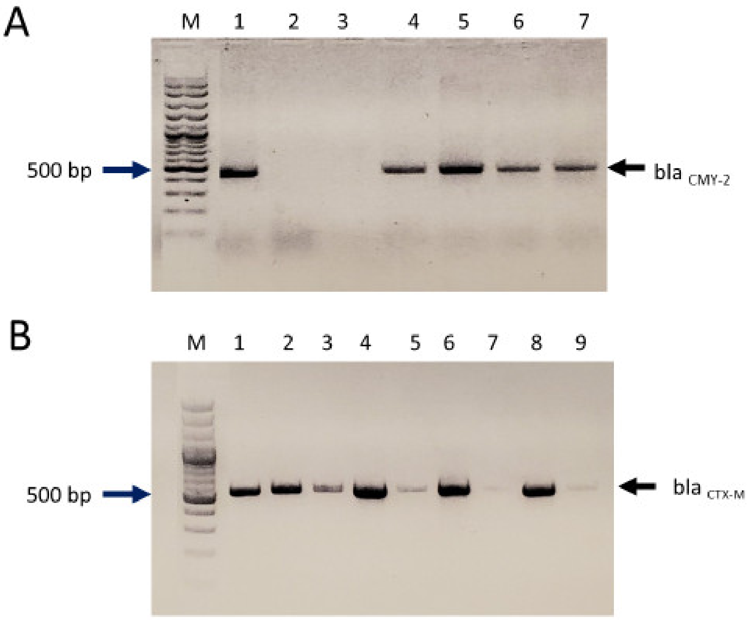

Figure S3. PCR products for bla-$_{\text{CMY-2}}$, 454 bp (**A**) or bla-$_{\text{ctx-M}}$, 554 bp (**B**), obtained from donor (D) ExPEC/APEC strains and corresponding trans-conjugants(Tc) following mating with *E. coli* K12^{NA}. **A.** Lanes 1-7 correspond to Positive Control (PC), F16.2 (D), F16.2 (Tc), F42.2 (D), F42.2 (Tc), F53.1 (D), F 53.1 (Tc), respectively. All trans-conjugants were selected on NA+Amp+Str **B.** Lanes 1-10 correspond to PC, F11.1 (D), F11.1 (Tc selected on NA+Str), F11.1(Tc selected on NA +Str+Amp), F15.2 (D), F15.2 (Tc from NA+ Amp), F15.2 (Tc from NA+Str+Amp), F16.2 (D), F16.2 (Tc), respectively. M is the GeneRuler 100 bp Plus DNA ladder (Thermoscientific).
